# How Algorithmic Management Influences Gig Workers’ Job Crafting

**DOI:** 10.3390/bs14100952

**Published:** 2024-10-15

**Authors:** Rong Liu, Haorong Yin

**Affiliations:** School of Business, Hohai University, Nanjing 211100, China; 19980044@hhu.edu.cn

**Keywords:** algorithmic management, gameful experience, perceived job autonomy, job crafting, core self-evaluation, social information processing theory

## Abstract

Algorithmic management, as a digital management tool in the gig economy, has become a hotspot of concern at the intersection of theory and practice. However, there is a lack of research on the mechanisms and boundary conditions through which algorithmic management affects gig workers’ job crafting. Based on the social information processing theory, this study constructed a dual-mediation model of how algorithmic management influences gig workers’ job crafting through gameful experience and perceived job autonomy. Data from 687 valid samples were collected through a two-stage survey and statistically analyzed using structural equation modeling (SEM). The results demonstrate that algorithmic management increases gig workers’ promotion-focused job crafting behaviors (increasing job resources and challenging job demands) by stimulating their gameful experiences and increases gig workers’ prevention-focused job crafting behaviors (decreasing hindering job demands) by inhibiting their perceived job autonomy. In addition, the higher-order personality trait core self-evaluation moderates the relationship between algorithmic management and gameful experience and perceived job autonomy, as well as the indirect effects of algorithmic management on job crafting through gameful experiences and perceived job autonomy. This study advances empirical research on algorithmic management in the field of the gig economy and human resource management practices. Crucially, it provides practical insights for optimizing algorithmic systems in platform companies, potentially enhancing their efficiency and economic benefits.

## 1. Introduction

Recent years have seen a rapid development of online labor platforms due to the advancement of digital technologies including big data, cloud computing, and smart terminals. The number of gig workers participating in such platforms’ services has continued to increase, and the gig economy has flourished [1,2]. The so-called gig workers of the digital age are workers who are flexibly employed by accepting orders through online labor platforms and then participating in offline services [3,4]. This new economy, characterized by flexible and multiple work tasks, has revolutionized the human resources management model, transforming traditional “offline” human resources management into “online” algorithmic management [5,6]. Algorithmic management is a new management tool in the digital age that operates on online labor platforms, providing autonomous and flexible work environments and precise and instant incentives that improve the platforms’ efficiency and economic benefits [7]. Algorithmic management is, therefore, reshaping the work environment and characteristics of gig workers in new and disruptive ways [8,9], and the rise of algorithmic booms has attracted extensive attention from academics.

Current research related to algorithmic management from the perspective of gig workers focuses on individual psychological, cognitive, and behavioral levels. For instance, some studies have pointed out that the opaqueness of decision-making in algorithmic management reduces gig workers’ perceptions of fairness [10,11]. Additionally, digital means of control continually diminish workers’ well-being [12] and exacerbate work anxiety [13], which in turn lowers their creativity and work performance [14,15]. However, a few scholars have argued that algorithmic management can enhance the affective experience and job satisfaction of gig workers at work through effective reward mechanisms [16,17]. Jarrahi and Sutherland (2019) found that gig workers adapt positively to algorithmic management based on the information feedback and work instructions provided by the algorithm and are also able to avoid algorithmic monitoring and capture vulnerabilities in the algorithmic system [18]. Thus, the impact of algorithmic management on an individual’s cognitive experience and work behavior is twofold [19,20]. However, due to the varying results of current studies, researchers must explore the mechanisms of algorithm management’s effects on gig workers’ attitudes and behaviors from a more comprehensive and systematic perspective.

Algorithms play the role of virtual managers in work situations with minimal human intervention, changing the way gig workers work and the working relationships between individuals and organizations [21]. The traditional “top-down” job design is no longer applicable, and platform companies need to rely on gig workers to take the initiative to personalize their job design, improve work efficiency, and promote long-term business growth [22]. Researchers defined this proactive coordination of job demands and resources for job redesign as a job crafting [23]. Furthermore, academics categorized job crafting into two types: promotion-focused job crafting and prevention-focused job crafting, based on the regulatory focus theory [24]. Therefore, studying how platforms can improve gig workers’ work experience through algorithmic management and trigger different types of job crafting is an important variable in the co-creation of value between gig workers and platforms, which has important theoretical value and practical significance.

According to the social information processing theory, individuals form or adjust their work attitudes and behaviors by interpreting and processing information about the characteristics of work in an organizational situation. This adjustment can be either negative or positive [25]. In the gig economy, algorithmic management serves as an important source of information that influences gig workers’ emotions and cognition by conveying different types of work information, which sequentially triggers corresponding behavioral changes. On the one hand, algorithmic systems create a gamified work atmosphere that conveys comprehensive and interesting task information [26], which pushes gig workers to have a positive, gameful experience and thus is more likely to be manifested in promotion-focused job crafting. On the other hand, the information conveyed by algorithmic management through management mechanisms such as strict monitoring, task assignment, and performance management makes gig workers perceive a lack of job autonomy [27]. Such negative perceptions may induce gig workers to adopt prevention-focused job crafting strategies.

Individuals are active decision-makers with significantly differing personality traits. This leads to individuals having different responses following selective information processing of the same situational cues [28]. In this study, we also seek to further explore which of the gig workers’ traits can enhance the positive and reduce the negative effects of algorithmic management. Core self-evaluation, as a higher-order personality trait, refers to an individual’s perceptions and judgments of their own abilities and values [29]. Specifically, gig workers with high core self-evaluations have positive judgments and cognitive dispositions toward situations in which they find themselves [30]. They are more attentive to the positive messages conveyed by algorithmic management and produce enjoyable, gameful experiences that are then implemented in promotion-focused job crafting. Moreover, gig workers with high core self-evaluations have a strong sense of personal control and self-regulation [31]. Therefore, the negative characteristics exhibited by algorithmic management do not pose much of a threat to such gig workers’ perceived job autonomy, thereby reducing their tendency toward prevention-focused job crafting.

Integrating the above arguments, this study builds a systematic dual-mediation model based on the social information processing theory. It follows the logical path of “information-cognition-behavior”, and empirically investigates this emerging algorithmic management mechanism. This study is intended to address three core questions: (1) whether algorithmic management can facilitate gig workers’ job crafting behaviors; (2) what the internal mechanism of the influencing process is; and (3) what the boundary conditions are for the positive effects of the dual-mediation mechanism of algorithmic management.

This paper comprises five sections. Section 1, the introduction, presents a comprehensive overview of the research, including its background, significance, and scope. In Section 2, we elaborate on the theoretical foundation of the study and present relevant definitions and the process of hypothesis derivation. Section 3 details the research design and the scales employed in the questionnaire survey. Section 4 explains how SPSS and AMOS were utilized to analyze the data and discusses the empirical results. Section 5 summarizes the research conclusions, contributions, limitations, and future prospects.

## 2. Theoretical Foundations and Research Hypotheses

### 2.1. Algorithmic Management and Job Crafting

The concept of job crafting originated with Kulik et al. (1987), who argued that “employees spontaneously redesign their jobs” [32]. Current research on job crafting is mainly based on Wrzesniewski et al.’s (2001) role view and Tims et al.’s (2010) resource view. In-depth comparisons revealed that both the role view and the resource view assert that job crafting has a positive impact on factors such as employees’ internal motivation and performance [23,33]. However, the studies by Rudolph et al. (2017) and Mkikangas et al. (2018) showed that reducing obstructive job requirements or downsizing work tasks were not positively associated with employees’ internal motivation and job performance [34,35]. Therefore, to rationalize this phenomenon, Lichtenthaler et al. (2018) employed the resource-based view to redefine job crafting by integrating regulatory focus theory and categorizing it into promotion-focused job crafting and prevention-focused job crafting [24]. According to this integrative concept, promotion-focused job crafting refers to remodeling behaviors that acquire positive job resources and outcomes with the goal of promotion, including increasing job resources and challenging job demands, while prevention-focused job crafting refers to remodeling behaviors that avoid negative job demands and outcomes with the goal of defense, including reducing obstructive job demands. This integrative concept more accurately and scientifically matches the two types of job crafting in terms of connotations and dimensions. Therefore, this study examines algorithmic management as a precursor influencing both promotion- and prevention-focused job crafting among gig economy workers.

Established research suggests that HRMS plays an important context-driven role in predicting job crafting behavior [33]. Algorithmic management is defined as an emerging organizational management tool that uses algorithmic technology to perform a range of management functions such as monitoring, goal setting, task assignment, performance rating, compensation management, job termination, and so on, instead of human managers [36,37]. Furthermore, according to the social information processing theory, algorithmic management serves as an important organizational cue, which can send gig workers either positive or negative job messages. In turn, these messages shaped their work behavior [38,39]. Therefore, we suggest that algorithmic management contributes to the job crafting of gig workers.

Specifically, gig workers use algorithmic management systems to receive effective information resources such as labor service-related job instructions and customer evaluations [40] to clarify which behaviors can be recognized by the platform. Therefore, they respond positively to the technical support provided by the algorithmic system and improve their work efficiency by learning new skills and accumulating work resources for promotion-focused job crafting. In addition, algorithmic systems update the performance ranking in real time on the work page and provide effective incentives, creating a work environment in which positive work can be rewarded by the platform and recognized favorably by coworkers, which can inspire the work enthusiasm of gig workers [41], strengthen work motivation, and stimulate the behavior of promotion-focused job crafting. Accordingly, the following hypothesis is proposed:

**Hypothesis** **1a.** *Algorithmic management is positively related to promotion-focused job crafting*.

Simultaneously, algorithmic systems provide continuous tracking and intervention based on technical rules and standardized workflows embedded in the platform [1]. The informational cues conveyed by these management measures create feelings of deprivation and job insecurity for gig workers [42], thereby negatively influencing their behavioral responses and inducing prevention-focused job crafting. Second, the algorithms manage to combine complex factors to automatically determine gig workers’ job ratings and pay [43]. This type of opaque decision-making can make gig workers feel that algorithmic management lacks respect for them, which typically results in negative attitudes [44] and an increase in prevention-focused job crafting, such as intentionally screening customer groups and ignoring algorithmic reminders [45,46].

In addition, the job demands–resources (JD-R) model categorizes job characteristics into two main categories: job demands and job resources [47]. Job demands refer to the individual physical and psychological resources that need to be consumed at work, such as workload, time pressure, serving demanding customers, uncomfortable scheduling, and unfavorable physical environments [48,49]. This theory posits that excessively high job demands can lead to burnout, resulting in negative work behaviors. Under the supervision of the algorithmic system, gig workers’ labor service process is completely exposed, and they are forced to constrain their work behaviors according to the algorithmic norms and accomplish unreasonable work goals by overloading their work, thus falling into the resource depletion dilemma. To handle this dilemma, the gig workers will adopt prevention-focused job crafting to protect their resources to reduce depletion. In conclusion, we propose the following hypothesis:

**Hypothesis** **1b.** *Algorithmic management is positively related to prevention-focused job crafting*.

### 2.2. The Mediating Role of Gameful Experience

Gamification refers to the use of game elements in non-game environments to create a game-like experience [50]. Eppmann et al. (2018) further pointed out that the gameful experience is a complex and multidimensional psychological structure that encompasses positive emotions such as enjoyment, absorption, and dominance when using gamified applications in non-game contexts [51]. Unlike the traditional human resource management model, work gamification in algorithmic management is an emerging management concept that involves the use of games to package work through the introduction of points, leaderboards, virtual identities, and other game elements [52], and the transformation of gig workers into “game players”, creating a gamified work atmosphere [53]. Previous studies have shown that gamified work design can promote positive cognition and emotions among employees [54,55]. Consequently, we argue that algorithmic management can be considered an informational cue that motivates gig workers to pursue gameful experiences.

On the one hand, the platform assigns tasks of different difficulties and rewards the gig workers based on algorithmic recommendations and sets up a task progress bar on the work page [41], in which the conveyed task information significantly activates the excitement and dominance of the gig workers, similar to that of completing a game task, thus generating a strongly gameful experience. On the other hand, algorithmic platforms provide instant virtual rewards, such as points, grades, and rankings. This positive information and effective incentives can motivate gig workers to eliminate negative emotions and generate a sense of achievement with timely satisfaction [56], which in turn creates gameful experiences. In summary, algorithmic management, through gamified work designs, enhances gig workers’ enjoyment and efficacy by providing task information and performance feedback [57], which is conducive to the creation of an immersive, gameful experience for them. In conclusion, we propose the following hypothesis:

**Hypothesis** **2.** *Algorithmic management is positively related to gameful experience*.

Previous studies have shown that a favorable gamification experience can lead to positive effects such as enhancing participants’ intrinsic motivation [58], stimulating employees’ creativity [59], and improving satisfaction [60,61]. According to the social information processing theory, an individual’s intrinsic reaction tendencies can often predict the occurrence of their actual behavior [25]. Therefore, following the logical view of “information input–individual emotion–work behavior”, we suggest that algorithmic management can influence the emotions of gig workers (generating a gameful experience) through input information (gamified work design). This emotional shift, in turn, promotes corresponding behavioral adjustments (promotion-focused job crafting). Specifically, when gig workers perceive a positive work experience resulting from algorithmic management, they will be more willing to proactively adjust to the work behaviors expected by the platform based on the information guidance provided by algorithms [62] and to improve their work skills to increase structural work resources. Second, the recognition and sense of competence that gig workers gain through ranking improvement on the leaderboard motivate them to be more willing to proactively share their work experiences and collaborate with their colleagues to complete their work tasks [57], consequently enhancing their individual social resources. In addition, gameful experience fits an individual’s challenge and competition psychology, inducing gig workers to actively participate in challenging tasks [63]. It is thus proposed that algorithmic management contributes to a purely gameful experience for gig workers during their labor process, increasing work motivation and engagement [64], which further enhances their promotion-focused job crafting. In conclusion, we propose the following hypothesis:

**Hypothesis** **3.** *Gameful experience mediates the relationship between algorithmic management and promotion-focused job crafting*.

### 2.3. The Mediating Role of Perceived Job Autonomy

As the impact of algorithmic management continues to deepen, it is likely to have potential negative effects along with positive effects on gig workers [65]. Hackman and Oldham (1976) defined perceived job autonomy as the degree to which employees perceive that they are able to make autonomous arrangements of work methods and standards in the performance of their work according to their own wishes [66]. The existing research suggests that the application of digital technology in the gig platform has a significant impact on perceived job autonomy [67,68]. Accordingly, we argue that algorithmic management implemented on online labor platforms can be considered an informational cue that inhibits gig workers’ perceived job autonomy.

First, in contrast to traditional normative work arrangements, algorithmic management automatically assigns work tasks and schedules based on big data. This mechanism leaves gig workers unable to predict their work schedules [69]. Faced with uncertain work schedules and environments due to algorithmic management, they feel a loss of control over their work, which reduces their perception of autonomy [70,71]. Second, real-time data updates in the algorithmic system lead to changes in performance metrics, and dynamic metrics stimulate overwork in the pursuit of a “high income” among gig workers [21,72]. This type of exploitation increases income uncertainty and reduces gig workers’ perceived autonomy [73]. Third, algorithms can automate decision-making based on quantitative metrics and directly fire gig workers [16]. This arbitrary decision-making creates an atmosphere of insecurity, undermining their perceived job autonomy [74]. In summary, the comprehensive regulation of algorithmic management weakens perceived job autonomy among gig workers, and we propose the following hypothesis:

**Hypothesis** **4.** *Algorithmic management is negatively related to perceived job autonomy*.

Recent studies have indicated that an increase in employees’ level of job autonomy is associated with a corresponding rise in their level of job crafting. Specifically, individuals who possess a stronger sense of self-perception are more likely to engage in job crafting activities [75]. According to the social information processing theory, individuals can make judgments by evaluating their information cues from the work social environment in order to adopt appropriate behaviors. Therefore, following the logic of “information input–individual cognition–work behavior”, we suggest that algorithmic management can influence the cognition (perceived job autonomy) of gig workers through the input of information (over-controlling information), which in turn induces them to make behavioral adjustments (prevention-focused job crafting) [25]. Specifically, strict monitoring and arbitrary decision-making in algorithmic management leads gig workers to infer that they do not receive recognition and protection of their rights and interests. This perception hinders their ability to appreciate the value of their work, resulting in a diminished sense of job autonomy [76]. This negative cognition prompts them to lower their work requirements and adopt prevention-focused job crafting. For example, in a high-intensity work environment, in order to avoid the depletion of physical and mental health caused by maintaining a highly stressful work state for a long period of time, gig workers will reasonably adjust their work strategies and time schedules, and systematically select peak ordering times and busy areas to optimize their work time and space [77]. Even experienced gig workers capture loopholes in the algorithmic system to manipulate orders [7] and work in a manner that is consistent with their experience to minimize the interference of algorithmic management in the work process [18]. Thus, algorithmic management reduces the perceived job autonomy of gig workers by implementing functions such as algorithmic monitoring, task assignment, and payroll management. This weakening of motivation ultimately contributes to a shift toward prevention-focused job crafting. In conclusion, we propose the following hypothesis:

**Hypothesis** **5.** *Perceived job autonomy mediates the relationship between algorithmic management and prevention-focused job crafting*.

### 2.4. The Moderating Role of Core Self-Evaluation

The concept of core self-evaluations was first proposed by Judge et al. (2003) as an individual’s self-perception, primarily encompassing an integrated assessment of one’s abilities, self-worth, and competence. Core self-evaluations represent a higher-order integration of four constructs: self-esteem, self-efficacy, emotional stability, and locus of control [29]. This concept expands and complements the Big Five personality theory and has been widely applied in research within the field of management psychology. Many scholars believe that core self-evaluations, as a potential personality trait, play a significant moderating role during employees’ work processes [78,79]. According to the social information processing theory, individual differences can influence how information is processed, leading to varying degrees of perception and behavioral adjustments [28]. This suggests that an individual’s cognitive state and behavioral reformation are not solely dependent on the external environment but are also related to individual characteristics. Even in identical algorithmic management contexts, gig workers with differing core self-evaluations may perceive the information relayed by the system differently, resulting in variability in the effectiveness of algorithmic management. Consequently, we predicted that core self-evaluation would influence the subjective constructs and evaluations of algorithmic management among gig workers, which would subsequently have a weighting effect on promotion-focused/prevention-focused job crafting.

Initially, individuals with a high level of core self-evaluation have a more optimistic attitude toward work-related matters and can actively seek rich meaning in their work [80], which can help gig workers better enjoy the process of gamification and increase their gameful experience. Additionally, gig workers who exhibit high core self-evaluations demonstrate greater enthusiasm for their roles and are inclined to set more challenging work goals [81,82]. Therefore, the social information released by algorithmic management that shapes the gamified work atmosphere is more likely to be recognized by gig workers with high core self-evaluations, resulting in enjoyable gameful experiences that subsequently proactively exhibit promotion-focused job crafting behaviors to acquire work resources and complete challenging tasks [83]. In contrast, when gig workers’ core self-evaluations are low, gamified work information released by algorithmic management fails to effectively stimulate their positive emotions, thus weakening the generation of gameful experiences and shaking their beliefs in proactively engaging in promotion-focused job crafting. Accordingly, we propose the following hypothesis:

**Hypothesis** **6a.** *Core self-evaluation strengthens the positive relationship between algorithmic management and gameful experience*.

**Hypothesis** **6b.** *Algorithmic management is positively indirectly related to promotion-focused job crafting through gameful experience*.

According to Judge et al. (2003), gig workers who maintain high core self-evaluations are “well-adjusted, positive, self-confident, effective, and believe in their own agency” [29]. They are able to positively perceive their competence and value and self-regulate their work resources. This self-regulation mitigates the perception of job non-autonomy often associated with algorithmic management [84]. Research has shown that individuals with high core evaluations tend to be more sensitive to positive stimuli, less sensitive to negative stimuli, and able to proactively adapt to the work environment [30]. Consequently, gig workers with high levels of core self-evaluation are less likely to be overly attentive to the negative messages conveyed by algorithmic management, which reduces the extent of negative perceptions of job autonomy and prevention-focused job crafting [85]. In contrast, gig workers with lower core self-evaluations are prone to exaggerate the negative information embedded in algorithmic management and develop stronger perceptions of a lack of job autonomy, thus increasing their likelihood of inducing prevention-focused job crafting. Accordingly, we propose the following hypothesis:

**Hypothesis** **7a.** *Core self-evaluation weakens the negative relationship between algorithmic management and perceived job autonomy*.

**Hypothesis** **7b.** *Algorithmic management is positively and indirectly related to prevention-focused job crafting through perceived job autonomy*.

**Figure 1:**
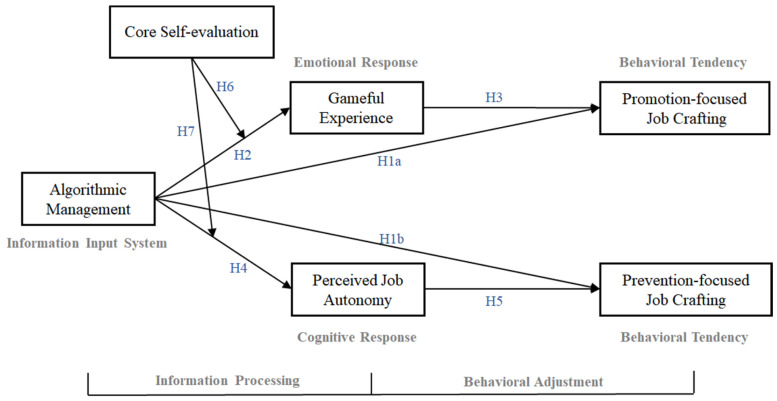
Research model.

In summary, this study proposes the theoretical model presented in Figure 1.

## 3. Materials and Methods

### 3.1. Sample and Procedures

This study focused on gig workers employed by digital platforms. Among the most representative and widely distributed groups within the gig economy, online drivers and delivery workers exhibit a high degree of dependence on these platforms for their employment. Consequently, they serve as a significant target for platform algorithmic management. Therefore, the sample for this study was restricted to two specific groups: online delivery workers and online car drivers who gain employment through online labor platforms.

This study adopted the questionnaire survey method. Questionnaires were distributed through WJX (https://www.wjx.cn/, a platform providing functions equivalent to Amazon Mechanical Turk). To incentivize the participants to fill out the questionnaires seriously and ensure the validity of the online questionnaires, the system provided the subjects with a certain amount of red packet rewards according to the content and length of the questionnaires filled out by the subjects. A total of 900 questionnaires were distributed in the first study (22 February–3 March 2024), and 819 valid questionnaires were returned (91% validity rate), including information on perceived algorithm management, perceived job autonomy, gameful experience, core self-evaluation, and demographic variables. The second study was conducted two weeks later (18–28 March 2024) and included responses on promotion- and prevention-focused job crafting. To ensure the proper tracking of the matched questionnaires, the gig workers filled in the last four digits of their cell phone numbers as codes for the tracking questionnaires. A total of 819 matched questionnaires were distributed, and questionnaires with irregular responses and those missing significant data were excluded, resulting in the retention of 687 valid matched questionnaires (valid recovery rate of 83.9%). Among the 687 completed questionnaires, 468 male workers accounted for 68.1%; ages less than 25 years old accounted for 20.2%; college education or below accounted for 82.5%; takeaway platforms accounted for 59.8%; full-time accounted for 77.9%; monthly income of more than 700 dollars accounted for 72.2%; average daily working hours more than 8 h accounted for 70.9%; working experience of more than 4 years accounted for 49.1%; and reasons for engaging in odd jobs were as follows: 20.4% for fun, 30.4% for important source of livelihood, 30.9% to subsidize family income, and 18.3% for flexibility and freedom.

### 3.2. Measurement

This study employed a questionnaire survey, the scale of which was defined according to the content of the study by drawing on the mature scale in previous studies, which has good reliability and validity. The questionnaire design adopted a five-point Likert scale ranging from 1 (totally non-conformity) to 5 (totally conformity). Higher scores reflect greater conformity levels. The questionnaire is presented in Appendix A.

Algorithmic management was measured using a 20-item scale developed by Parent-Rocheleau et al. [82]. One sample item was, “The algorithm management system closely monitors me while I am working”.

Gameful experience was measured using a 27-item scale developed by Eppmann et al. [51]. One sample item was, “Gameful experience at work is enjoyable”.

Perceived job autonomy was measured using a 7-item scale developed by Shirom [86]. One sample item was, “I am free to decide what to do during working hours”.

Promotion-focused job crafting was measured using a 15-item scale developed by Tims et al. [87]. One sample item was, “I try to develop my abilities at work”.

Prevention-focused job crafting was measured using a 6-item scale developed by Tims et al. [87]. One sample item was, “I try to keep myself from doing things at work that are difficult to choose”.

Core self-evaluation was measured using a 12-item scale developed by Judge et al. [29]. One sample item was, “I am confident that I will have the success I deserve”.

This study utilized the statistical characteristic variables as control variables, specifically including gig workers’ gender, age, education level, monthly income, and years of working experience. In addition, factors related to average daily working hours, type of platform (takeaway platform, online car platform), form of work (full-time, part-time), and reason for work (fun, important source of livelihood, subsidizing household, flexible and free) were selected to be controlled in light of the specificities of gig work.

## 4. Analysis and Results

This research utilized AMOS 24.0 and SPSS 26.0 software to validate the data. Given the extensive number of problematic question items for the study variables, the study modeled the multidimensional variables with internal indicator packing in order to improve the quality of the indicators and the goodness of fit of the model [88].

### 4.1. Confirmatory Factor Analysis

In this study, confirmatory factor analyses were conducted using AMOS 24.0 to test the validity of the model [89]. Table 1 shows the model fit indices for which the six-factor model fits optimally (χ^2^/df = 1.817, RMSEA = 0.035, SRMR = 0.035, CFI = 0.958, TLI = 0.955, IFI = 0.958). This indicated good structural and discriminant validity between the variables.

### 4.2. Common Method Variance

Although a longitudinal study design was used to minimize the adverse effects of common method bias on the findings, it was necessary to conduct a homogeneity bias test because the questionnaire was self-reported by gig workers [90]. The test was conducted using the Harman single-factor test in SPSS software by placing all the question items of the study variables into an exploratory factor analysis at the same time. The results of the unrotated factor analysis showed that the total variance explained by all the factors with an eigenroot greater than 1 was 65.661%, with the first one having a variance explained of 23.737%, which is less than 40%. This finding indicates that common method bias remains within an acceptable range, allowing further analysis in subsequent steps.

### 4.3. Descriptive Statistics, Correlation Coefficients, and Reliability and Validity Analysis

Table 2 presents the descriptive statistical analysis, correlation coefficients, and reliability test indicators of the variables in this study. As shown in Table 2, the Cronbach’s α coefficients of all constructs in this study are greater than the critical value of 0.7, and the combined reliability CR is much greater than the critical value of 0.5, which has a good reliability. The scales used in the study were all based on mature scales in the existing literature, ensuring good content validity. The AVE values of all constructs were greater than the critical value of 0.5, which had good convergent validity. The square root of the AVE for all constructs was greater than the correlation coefficient between the variables, so the scales had good discriminant validity [91].

The preliminary analysis of the correlation coefficients showed that algorithmic management was significantly positively correlated with both gameful experience (r = 0.512, *p* < 0.01) and promotion-focused job crafting (r = 0.444, *p* < 0.01) and that gameful experience was significantly positively correlated with promotion-focused job crafting (r = 0.455, *p* < 0.01). Algorithmic management was significantly negatively correlated with perceived job autonomy (r = −0.385, *p* < 0.01) and significantly positively correlated with prevention-focused job crafting (r = 0.404, *p* < 0.01), whereas perceived job autonomy was significantly negatively correlated with prevention-focused job crafting (r = −0.344, *p* < 0.01). The results of the descriptive statistics and correlation analyses preliminarily validate the subsequent hypotheses.

### 4.4. Hypothesis Testing

The hypotheses were tested using a regression analysis [92]. As the reason for working is a categorical variable, it was treated as a dummy variable with interest as the reference group. The significance of the mediation effect and moderated mediation were also estimated using the Process 4.1 plug-in, the bias-corrected nonparametric percentile Bootstrap method, where the Bootstrap was 5000 times.

#### 4.4.1. Main and Mediating Effects Analysis

Hierarchical regression was used to statistically test the hypotheses, and the results are shown in Table 3. From Model 2 and Model 5, it can be seen that algorithmic management had a significant positive effect on promotion-focused job crafting (β = 0.265, *p* < 0.001) and a significant positive effect on prevention-focused job crafting (β = 0.313, *p* < 0.001), so H1a and H1b were both valid.

First, regression analysis was conducted on gameful experience and algorithm management, and from M1, algorithm management had a significant positive effect on gameful experience (β = 0.354, *p* < 0.001). Therefore, hypothesis H2 was verified. Secondly, adding the mediating variable gameful experience based on M2 to reach M3, the results show that algorithm management still had a significant positive influence on promotion-focused job crafting (β = 0.175, *p* < 0.001); however, the degree of the influence was reduced, and the gameful experience had a significant positive influence on promotion-focused job crafting (β = 0.255, *p* < 0.001), indicating that the gameful experience had a partial mediating effect in algorithmic management and promotion-focused job crafting, and that hypothesis H3 was valid. Similarly, the regression analysis of perceived job autonomy and algorithmic management showed a significant negative effect of algorithmic management on perceived job autonomy from M4 (β = −0.229, *p* < 0.001), and hypothesis H4 was established. Adding the mediator variable job autonomy perception based on M5 to reach M6, the results show that algorithm management also had a significant positive effect on prevention-focused job crafting (β = 0.269, *p* < 0.001), with the degree of influence decreasing. In addition, job autonomy perception had a significant negative effect on prevention-focused job crafting (β = −0.193, *p* < 0.001), indicating that job autonomy perception had a partial mediating effect in algorithmic management and prevention-focused job crafting, as preliminarily verified in H5.

To further verify H3 and H5, the Bootstrap method was used to test the robustness of the mediating effect [93]. The mediating effects of gameful experience and perceived job autonomy in the model were tested using the plug-in PROCESS 4.1 for SPSS, with a sample size of 5000 and a 95% confidence interval. The results of the run indicate that the mediating effects of gameful experience and perceived job autonomy were 0.090 and 0.044, respectively, and the 95% confidence intervals were asked [0.059, 0.124] and [0.022, 0.068], respectively, which did not contain a 0. Therefore, H3 and H5 were validated.

#### 4.4.2. Moderating Effects Analysis

First, the moderating effect of core self-evaluation was tested using a stratified regression, and the results are shown in Table 4. As shown in Model 8, there was a significant positive effect of the interaction term of algorithmic management and core self-evaluation on gameful experience (β = 0.126, *p* < 0.001). As shown in Model 10, the interaction term of algorithmic management and core self-evaluation on perceived job autonomy had a significant positive effect (β = 0.077, *p* < 0.01). Thus, H6a and H7a were tentatively supported.

**Figure 2:**
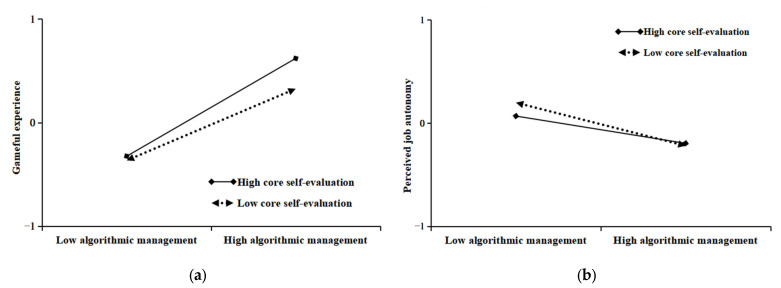
Moderating effect. (**a**) Moderating effect of CSE on relationship between AM and GE. (**b**) Moderating effect of CSE on relationship between AM and PJA.

To more intuitively represent the moderating effect of core self-evaluation, we referred to the simple slope analysis procedure of Toothaker [94]. The two core self-evaluation levels were divided into high and low groups according to one standard deviation above and one standard deviation below the mean. They were regressed separately to draw a moderating effect graph to show the direction and intensity of the moderating effect more intuitively. As presented in Figure 2a, the slope of the regression straight line for high core self-evaluation is larger than that for low core self-evaluation, indicating that the same intensity of algorithmic management had a relatively larger facilitating effect on the gameful experience of gig workers when the level of core self-evaluation was higher. Meanwhile, Figure 2b illustrates that the slope of the regression straight line for high core self-evaluation is smaller than the slope of the regression straight line for low core self-evaluation, indicating that when the level of core self-evaluation was low, the same intensity algorithmic management had a relatively larger role in weakening the perceived job autonomy of the gig workers’ work. Therefore, there was a significant difference in the moderating effect at high and low levels, and hypotheses H6a and H7a were again tested.

#### 4.4.3. Moderated Mediation Effects Analysis

The moderated mediating role of core self-evaluation in the model was tested using the SPSS add-in PROCESS 4.1, with a sample size of 5000 and a bias-corrected confidence interval of 95% [95]. As shown in Table 5, the mediating effect of gameful experience was significant in the high core self-evaluation condition [β = 0.120, CI (0.080, 0.165)] and in the low core self-evaluation condition [β = 0.056, CI (0.027, 0.088)], and the difference between the mediating effects in the two conditions mentioned above was significant (CI [ 0.031, 0.108]). Thus, core self-evaluation moderated the mediating effect of gameful experience, and H6b was valid. Similarly, the mediating effect of perceived job autonomy was significant in the high core self-evaluation condition [β = 0.025, CI (0.011, 0.055)] and in the low core self-evaluation condition [β = 0.055, CI (0.028, 0.085)], and the difference between the mediating effects was significant between the two conditions (CI [−0.058, −0.005]). Thus, core self-evaluation moderated the mediating effect of perceived job autonomy, and H7b was valid.

## 5. Discussion

Algorithmic management has been a hot topic in recent years in the gig economy and human resource management. The results of this study are as follows:

(1) This study investigates algorithmic management as an information source and validates its positive influence on both promotion-focused and prevention-focused job crafting, with individual emotions and cognition playing a mediating role. Specifically, when gig workers perceive the gameful experience brought by algorithmic management, they tend to reinforce promotion-focused job crafting; on the contrary, when gig workers perceive that algorithmic management restricts their job autonomy, they tend to increase prevention-focused job crafting. Previous research primarily focused on the negative impacts of algorithmic management [12,13], with limited attention given to its positive dimensions [16]. This study, grounded in the social information processing theory, analyzes the impact of algorithmic management on work redesign through these two pathways, thereby acknowledging the limitations of algorithmic management while also emphasizing its beneficial aspects and offering a more comprehensive and dialectical perspective on the role of algorithmic management.

(2) Core self-evaluation and algorithmic management interact to influence gig workers’ cognitive emotions, which in turn affects their job crafting. Specifically, core self-evaluation enhances the positive effect of algorithmic management on gameful experience and weakens the negative effect of algorithmic management on perceived job autonomy. At high levels of core self-evaluation, algorithmic management demonstrates a more significant positive effect on promotion-focused job crafting via gameful experiences. Conversely, at low levels of core self-evaluation, algorithmic management exhibits a more pronounced positive influence on prevention-focused job crafting through perceived job autonomy. These findings highlight that core self-evaluation serves as an important moderator on the relationship between individual cognition and behavior, further supporting the premise of its moderating role.

### 5.1. Theoretical Implications

First, this study enriches the job crafting research system and broadens the concept of job crafting to encompass the gig economy based on the dual-action path of algorithmic management. This marks a first-time investigation into the effect of algorithmic management on the job crafting behaviors of gig workers, which deepens the antecedent research on job crafting in the field of the gig economy. In addition, most related studies have not comprehensively examined the dimensions of job crafting, focusing mainly on promotion-focused job crafting and ignoring prevention-focused job crafting [96]. This research integrates the two dimensions of job crafting and explores the corresponding antecedent variables separately. Furthermore, previous research has predominantly explored the mechanisms of job crafting through various theoretical frameworks such as the job demands–resources model [97], conservation of resources theory [98], and self-determination theory [99]. In contrast, this study utilizes the logic of the social information processing theory to analyze job crafting along the pathways of information input, processing, and output, thereby expanding the theoretical foundation in the field of job crafting research.

Second, this study expands the research perspective on the mediating mechanisms of algorithmic management effectiveness. Currently, academic exploration of algorithmic management’s impact mechanisms remains in its early phases. Although existing studies have theoretically proposed the efficacy of the “hybridity” perspective of algorithmic management [7], the specific ways in which algorithmic management dynamically and complexly influences the work outcomes of gig workers require further exploration within concrete management contexts. The social information processing theory provides a suitable theoretical perspective for this investigation, allowing us to examine the dual effects of algorithmic management. From both emotional and cognitive viewpoints, we investigated the “black box” between algorithmic management and promotion-focused/prevention-focused job crafting behaviors, thus advancing the empirical research on the influence mechanism of algorithmic management.

Finally, core self-evaluation is an important condition for algorithmic management. Although existing studies generally recognize the importance of employees’ core self-evaluation of their work ability and motivation, they seldom consider it in a specific management context. This study examines and validates the moderating effect of core self-evaluation among gig workers and further clarifies its impact on the relationship between algorithmic management and job crafting. The findings help to answer the important question of when to promote the positive effects of algorithmic management and avoid the negative ones. This study not only offers new insights into how individuals can manage the impacts of algorithmic management but also expands the scope of research on core self-evaluation in organizational management.

### 5.2. Managerial Implications

First, platform companies should endeavor to enhance the beneficial outcomes of their algorithms. It is imperative that platforms consider the factors that can motivate gig workers to improve their algorithm systems. For example, by continuously optimizing the algorithm mechanism, the platform can deliver more comprehensive, transparent, and convenient task information support to gig workers. Additionally, it can enhance the sense of organizational support by integrating gamification elements and maximizing the guiding effect of game elements on the task behavior of gig workers to improve the sense of fun and their work experience.

Second, platform firms must mitigate the negative effects of their algorithmic management. The primary factor contributing to the demotivation of gig workers is the lack of autonomy in their work, which is a consequence of the excessive control exerted by algorithms. Therefore, more humanistic care should be incorporated into the process of algorithm management, and the subject position of gig workers should be respected. For example, the platform could provide a smooth complaint channel in process management to effectively solve contradictory problems at work; involve gig workers in the process of strategy formulation to enhance their sense of agency [100]; and eliminate the “strictest algorithm” as the assessment standard.

Third, it is recommended that platform enterprises adopt different management strategies for individuals with different CSE levels. Gig workers with high core self-evaluations are able to make timely self-adjustments and actively adapt to algorithmic management, whereas gig workers with low core self-evaluations lack stress resistance and respond negatively to algorithmic management. Therefore, platform enterprises are advised to conduct scientific assessments of individuals’ core self-evaluation levels at the recruitment and selection stages and carry out targeted training. In addition, the platform can establish a sound incentive mechanism to positively recognize gig workers’ performance to improve their core self-evaluation level and consciously help them establish a positive mindset. Simultaneously, it is essential to establish a comprehensive self-evaluation system for employees, enabling gig workers to gain a deeper understanding of themselves in order to make corresponding improvements and enhance their work performance.

### 5.3. Limitations and Future Research

This study has limitations that should be addressed in future studies.

In terms of research methodology, to minimize the adverse impact of the common method bias problem on the scientificity and reliability of the inferred conclusions, this study traced and collected data from multiple time points to improve the persuasiveness of the inferred causal relationships between variables. However, the research design of a single data source was still unable to fully circumvent the potential for common method bias which may be brought about by the self-assessment of gig workers. The big data-driven platform terminal collects a huge amount of data information about the daily working status of gig workers, such as customer evaluation data, daily income data, and effective working hours. The objective data accumulated by the platform can be leveraged to enhance the objectivity and accuracy of the study. Future research should utilize big data platforms to consider a more diverse range of data and longitudinal study designs to further investigate the relationships between variables.

In terms of the selection of research subjects, this study explores the utility of algorithmic management with low-skilled workers such as takeaway workers and online car drivers as the object of study. With the development of the Internet, currently the gig economy is no longer confined to low-skilled workers, and more and more knowledge-based talents are also engaged in high-skilled gig work. Further investigation is required to gain a deeper understanding of the ways in which algorithmic management affects the job crafting of knowledge-based gig workers, and to identify similarities and differences between the findings of this study.

While this study has examined the mechanism through which algorithmic management impacts job crafting based on the information processing and transmission principles of the social information processing theory, there are additional, more complex factors in the transmission path between the two that warrant further exploration. Future research should seek to develop new theoretical perspectives to enhance our understanding of how algorithmic management exerts its effects. For instance, the Technology Acceptance Model could be utilized to examine whether employees are willing to accept and use algorithmic tools, thereby actively engaging in job crafting; or the self-determination theory could be employed to explore how algorithms drive gig workers to adjust their intrinsic motivations and pursue proactive restructuring.

With regard to the selection of variables, because of the dual mechanism of the impact of algorithmic management on job crafting, identifying the moderating variables in which the dual effect plays a role is more valuable for balancing algorithmic management tensions. Future research could thus select moderating variables that better reflect the dual effect, such as goal orientation (learning/avoidance), control focus (internal/external), and job stress (challenging/hindering) for an in-depth exploration. Furthermore, although this study identifies certain control variables by integrating the unique characteristics of gig work, future research should explore more in-depth potential control variables to enhance the reliability of the findings, such as cultural differences and levels of technology acceptance.

## Figures and Tables

**Table 1:** Confirmatory factor analyses.

Models	χ^2^	df	Δχ^2^/df	RMSEA	SRMR	CFI	TLI	IFI
Six-factor model	1248.483	687	1.817	0.035	0.035	0.958	0.955	0.958
AM, PJA, GE, PROJC, PREJC, CSE								
Five-factor model	1996.232	692	2.885	0.052	0.069	0.903	0.896	0.903
AM, PJA, GE, CSE, PROJC + PREJC								
Four-factor model	3366.389	696	4.837	0.075	0.124	0.801	0.788	0.802
AM + CSE, PJA, GE, PROJC + PREJC								
Three-factor model	4477.103	699	6.405	0.089	0.133	0.719	0.702	0.72
AM + CSE, PJA + GE, PROJC + PREJC								
Two-factor model	6926.355	701	9.881	0.114	0.176	0.536	0.51	0.538
AM + CSE + PJA + GE, PROJC + PREJC								
One-factor model	8568.908	702	12.206	0.128	0.151	0.414	0.382	0.416
AM + CSE + PJA + GE + PROJC + PREJC								

N = 687; AM, algorithmic management; PJA, perceived job autonomy; GE, gameful experience; PROJC, promotion-focused job crafting; PREJC, prevention-focused job crafting; CSE, core self-evaluation.

**Table 2:** Descriptive statistics, correlation coefficients, and reliability and validity analysis of the main research variables.

Variable	M	SD	1	2	3	4	5	6	Cronbach’s α	CR	AVE
**1. AM**	3.799	0.611	0.766						0.923	0.966	0.587
**2. GE**	3.822	0.625	0.512 **	0.787					0.944	0.978	0.620
**3. PJA**	2.520	0.706	−0.385 **	−0.407 **	0.711				0.876	0.877	0.506
**4. CSE**	3.844	0.719	0.157 **	0.205 **	−0.139 **	0.717			0.926	0.927	0.514
**5. PROJC**	3.893	0.636	0.444 **	0.480 **	−0.443 **	0.183 **	0.769		0.919	0.956	0.591
**6. PREJC**	3.927	0.953	0.404 **	0.455 **	−0.344 **	0.192 **	0.286 **	0.795	0.911	0.911	0.632

* *p* < 0.05, ** *p* < 0.01, *** *p* < 0.001; on the diagonal is the square root of each variable AVE; AM, algorithmic management; PJA, perceived job autonomy; GE, gameful experience; PROJC, promotion-focused job crafting; PREJC, prevention-focused job crafting; CSE, core self-evaluation.

**Table 3:** Results of main effects and mediation effects.

Variable	Gameful Experience	Promotion-Focused Job Crafting		Perceived Job Autonomy	Prevention-Focused Job Crafting	
	Model 1	Model 2	Model 3	Model 4	Model 5	Model 6
Gender	−0.084	0.033	0.055	−0.039	−0.093	−0.101
Age	0.015	0.035	0.031	−0.016	0.023	0.02
Education	−0.029	0.022	0.029	−0.023	−0.079 *	−0.084 *
Platform type	0.008	−0.008	−0.01	−0.013	0.056	0.054
Form of work	−0.092	−0.214 ***	−0.191 ***	0.037	0.004	0.011
Income	0.208 ***	0.128 ***	0.075 *	−0.174 ***	0.057	0.023
Working hours	0.129 ***	0.201 ***	0.168 ***	−0.250 ***	0.143 ***	0.095 *
Years of work	0.009	0.042	0.04	0.042	−0.013	−0.005
Source of livelihood	0.061	0.059	0.044	0.015	0.024	0.027
Subsidizing family	0.061	0.014	−0.001	−0.002	0.026	0.026
Flexibility	0.025	0.008	0.002	−0.032	0.031	0.024
AM	0.354 ***	0.265 ***	0.175 ***	−0.229 ***	0.313 ***	0.269 ***
GE			0.255 ***			
PJA						−0.193 ***
R^2^	0.358	0.3	0.342	0.249	0.203	0.231
ΔR^2^	0.092 ***	0.052 ***	0.042 ***	0.038 ***	0.072	0.028
F	31.264 ***	24.036 ***	26.848 ***	18.638 ***	14.339 ***	15.582 ***

* *p* < 0.05, ** *p* < 0.01, *** *p* < 0.001.

**Table 4:** Results of moderating effects.

Variable	Gameful Experience		Perceived Job Autonomy	
	Model 7	Model 8	Model 9	Model 10
Gender	−0.084	−0.084	−0.039	−0.038
Age	0.017	0.021	−0.016	−0.014
Education	−0.036	−0.035	−0.019	−0.018
Platform type	0.007	0.005	−0.013	−0.014
Form of work	−0.099	−0.109 *	0.041	0.034
Income	0.209 ***	0.209 ***	−0.175 ***	−0.175 ***
Working hours	0.123 ***	0.136 ***	−0.247 ***	−0.239 ***
Years of work	0.004	−0.001	0.045	0.042
Source of livelihood	0.063	0.058	0.013	0.01
Subsidizing family	0.065	0.064	−0.004	−0.005
Flexibility	0.032	0.024	−0.036	−0.041
AM	0.332 ***	0.346 ***	−0.217 ***	−0.209 ***
CSE	0.135 ***	0.167 ***	−0.073 *	−0.053
AM*CSE		0.126 ***		0.077 **
R^2^	0.375	0.396	0.254	0.262
ΔR^2^	0.017 ***	0.021 ***	0.005 *	0.008 **
F	31.066 ***	31.496 ***	17.653 ***	17.070 ***

* *p* < 0.05, ** *p* < 0.01, *** *p* < 0.001.

**Table 5:** Indirect effects.

Variable	Indirect Effect	SE	Boot95%CI	
			Lower	Upper
Mediating variable: gameful experiences				
High core self-evaluation (+1SD)	0.120	0.022	0.08	0.165
Low core self-evaluation (−1SD)	0.056	0.016	0.027	0.088
Discrepancy	0.064	0.020	0.031	0.108
Mediating variable: perceived job autonomy				
High core self-evaluation (+1SD)	0.025	0.011	0.004	0.048
Low core self-evaluation (−1SD)	0.055	0.014	0.028	0.085
Discrepancy	−0.030	0.014	−0.058	−0.005

## Data Availability

The raw data supporting the conclusions of this article will be made available by the corresponding author upon reasonable request.

## Appendix A

The following are the measurement items adopted.

**Table A1:** Algorithmic management scale.

Items	Questionnaire Items
**Monitoring**	An automated system tracks me carefully to ensure I am completing my tasks.
	An automated system closely monitors me while I am doing my work.
	An automated system inspects my work closely.
	I am constantly being watched by an automated system to see that I obey the rules pertaining to my job.
**Goal setting**	My daily tasks are assigned by an automated system.
	An automated system decides what tasks I will be doing.
	In my job, an automated system determines what needs to be done.
	An automated system determines the targets I must attain at work (productivity targets, time targets, sales target, etc.).
	The targets I have to reach are set by the automated system.
**Scheduling**	An automated system decides when I work and when I do not.
	My work schedule is made by an automated system.
	An automated system is responsible for determining my working hours.
	My working hours are determined automatically by an electronic system.
**Performance rating**	The evaluation of my work performance is handled by an electronic system.
	An automated system generates the metrics used to assess my performance.
	My performance evaluation is based on metrics computed by an automated system.
**Compensation**	A large part of my compensation is determined by an automated system.
	The decisions related to my earnings are mostly made by the automated system.
	An automated system is responsible for calculating my pay, with no human intervention.
	What I earn is the result of an automated system calculation only.

**Table A2:** Gameful experience scale.

Items	Questionnaire Items
**Enjoyment**	Participating in platform gamification was fun.
	I liked participating in platform gamification.
	I enjoyed participating in platform gamification very much.
	My gameful experience was pleasurable.
	I think participating in platform gamification is very entertaining.
	I would participate in platform gamification for it own sake, not only when being asked to.
**Absorption**	Participating in platform gamification made me forget where I am.
	I forgot about my immediate surroundings while I participated in platform gamification.
	After participating in platform gamification, I felt like coming back to the “real world” after a journey.
	While participating in platform gamification “got me away from it all”.
	While participating in platform gamification I was completely oblivious to everything around me.
	While participating in platform gamification I lost track of time.
**Creative thinking**	Participating in platform gamification sparked my imagination.
	While participating in platform gamification I felt creative.
	While participating in platform gamification I felt that I could explore things.
	While participating in platform gamification I felt adventurous.
**Activation**	While participating in platform gamification I felt activated.
	While participating in platform gamification I felt jittery.
	While participating in platform gamification I felt frenzied.
	While participating in platform gamification I felt excited.
**Absence of** **negative**	While participating in platform gamification I felt upset.
	While participating in platform gamification I felt hostile.
	While participating in platform gamification I felt frustrated.
**Dominance**	While participating in platform gamification I felt dominant.
	While participating in platform gamification I felt influential.
	While participating in platform gamification I felt autonomous.
	While participating in platform gamification I felt confident.

**Table A3:** Perceived job autonomy scale.

Items	Questionnaire Items
**Perceived job** **autonomy**	I had freedom to decide what to do.
	I had freedom to decide how to do my own work.
	I had responsibility for deciding how the job got done.
	I had a lot to say about what happened on the job.
	I had latitude to decide when to take breaks.
	I had freedom to decide who I want to work with.
	had freedom to decide the speed of my work.

**Table A4:** Promotion-focused job crafting scale.

Items	Questionnaire Items
**Increasing structural job resources**	I try to develop my capabilities.
	I try to develop myself professionally.
	I try to learn new things at work.
	I make sure that I use my capacities to the fullest.
	I decide on my own how I do things.
**Increasing social job resources**	I ask my supervisor to coach me.
	I ask whether my supervisor is satisfied with my work.
	I look to my supervisor for inspiration.
	I ask others for feedback on my job performance.
	I ask colleagues for advice.
**Increasing challenging job demands**	When an interesting project comes along, I offer myself proactively as project co-worker.
	If there are new developments, I am one of the first to learn about them and try them out.
	When there is not much to do at work, I see it as a chance to start new projects.
	I regularly take on extra tasks even though I do not receive extra salary for them.
	I try to make my work more challenging by examining the underlying relationships between aspects of my job.

**Table A5:** Prevention-focused job crafting scale.

Items	Questionnaire Items
**Decreasing hindering job demands**	I make sure that my work is mentally less intense.
	I try to ensure that my work is emotionally less intense.
	I manage my work so that I try to minimize contact with people whose problems affect me emotionally.
	I organize my work so as to minimize contact with people whose expectations are unrealistic.
	I try to ensure that I do not have to make many difficult decisions at work.
	I organize my work in such a way to make sure that I do not have to concentrate for too long a period at once.

**Table A6:** Core self-evaluation scale.

Items	Questionnaire Items
**Core self-evaluation**	I am confident I get the success I deserve in life.
	Sometimes I feel depressed.
	When I try, I generally succeed.
	Sometimes when I fail I feel worthless.
	I complete tasks successfully.
	Sometimes, I do not feel in control of my work.
	Overall, I am satisfied with myself.
	I am filled with doubts about my competence.
	I determine what will happen in my life.
	I do not feel in control of my success in my career.
	I am capable of coping with most of my problems.
	There are times when things look pretty bleak and hopeless to me.
